# The Development of a Multilingual Tool for Facilitating the Primary-Specialty Care Interface in Low Resource Settings: the MSF Tele-Expertise System

**DOI:** 10.3389/fpubh.2014.00126

**Published:** 2014-08-26

**Authors:** Laurent Bonnardot, Joanne Liu, Elizabeth Wootton, Isabel Amoros, David Olson, Sidney Wong, Richard Wootton

**Affiliations:** ^1^Médecins Sans Frontières, Paris, France; ^2^Department of Medical Ethics and Legal Medicine (EA 4569), Paris Descartes University, Paris, France; ^3^Médecins Sans Frontières, Geneva, Switzerland; ^4^McGill University, Montréal, QC, Canada; ^5^University of Edinburgh, Edinburgh, UK; ^6^Médecins Sans Frontières, Barcelona, Spain; ^7^Médecins Sans Frontières, New York, NY, USA; ^8^Médecins Sans Frontières, Amsterdam, Netherlands; ^9^Norwegian Centre for Integrated Care and Telemedicine, University Hospital of North Norway, Tromsø, Norway; ^10^Faculty of Health Sciences, University of Tromsø, Tromsø, Norway

**Keywords:** telemedicine, telehealth, developing countries, tele-expertise, multilingual network

## Abstract

In 2009, Médecins Sans Frontières (MSF) started a pilot trial of store-and-forward telemedicine to support field workers. One network was operated in French and one in English; a third, Spanish network was brought into operation in 2012. The three telemedicine pilots were then combined to form a single multilingual tele-expertise system, tailored to support MSF field staff. We conducted a retrospective analysis of all telemedicine cases referred from April 2010 to March 2014. We also carried out a survey of all users in December 2013. A total of 1039 referrals were received from 41 countries, of which 89% were in English, 10% in French, and 1% in Spanish. The cases covered a very wide range of medical and surgical specialties. The median delay in providing the first specialist response to the referrer was 5.3 h (interquartile range 1.8, 16.4). The survey was sent to 294 referrers and 254 specialists. Of these, 224 were considered as active users (41%). Out of the 548 users, 163 (30%) answered the survey. The majority of referrers (79%) reported that the advice received via the system improved their management of the patient. The main concerns raised by referrers and specialists were the lack of support or promotion of system at headquarters’ level and the lack of feedback about patient follow-up. Because of the size of the MSF organization, it is clear that there is potential for further organizational adoption.

## Introduction

Médecins Sans Frontières (MSF) is an international, independent, and medical humanitarian organization that responds to emergency situations and provides medical assistance to people in need affected by armed conflict, epidemics, natural disasters, and exclusion from healthcare (1). A defining characteristic of the organization is its innovation (2). Over the years, MSF has developed considerable expertise in pioneering new technology for resource-limited settings in different fields, such as medical (e.g., automated TB diagnostic testing (GeneXpert), malaria Rapid Diagnostic Test) or logistical (e.g., inflatable hospitals with operating theaters, oxygen concentrators, vaccination kit).

It is not surprising, then, that MSF should take advantage of new information technology to improve the quality of health care for patients in low-resource settings. The work in question began in 2009, when MSF started a pilot trial of two telemedicine systems to support field workers. One was operated in French and one in English; a third, Spanish system was brought into operation in 2012. They were established initially in collaboration with the Swinfen Charitable Trust (3). In late 2013, the three telemedicine pilots were combined into a single multilingual system, using technology based on the Collegium Telemedicus system (4). Because of the constraints of MSF operations (e.g., legal, confidentiality, reporting), the multilingual system was established on a secure web server of its own, telemed.msf.org.

The product of this 4-year development period is a tele-expertise system, tailored to support MSF field staff. It is based on a highly secure web messaging system (see Box 1). It aims to facilitate the primary-specialty care interface by allowing a primary care physician to obtain an expert second opinion about a difficult clinical problem within a few hours.

Box 1 **The MSF tele-expertise system.**

**Purpose**

A tool for use in the field to improve access to specialized clinical advice. It is available in English, French, and Spanish.

**Workflow**

(1) Referrer logs in at https://telemed.msf.org using any web browser. Then submits a clinical case, including attachments if appropriate (e.g., pictures, video clips).
(2) Case-coordinator reviews the referral and allocates the case to an appropriate specialist. If there is no answer within 24 h, the case-coordinator re-allocates the case to another specialist.
(3) Specialist is notified by email that there is a referral requiring advice, logs in, and answers the case. The specialist can conduct a direct dialog with the referrer if required.

**Method of operation**

A secure, web-based messaging system. Confidentiality is ensured by removing any identifying patient data. Email is only used for notifications (i.e., that a case has been submitted or an answer received) and for advisory messages (e.g., login reminder).

**Example**

Clinical case sent through MSF tele-expertise system.

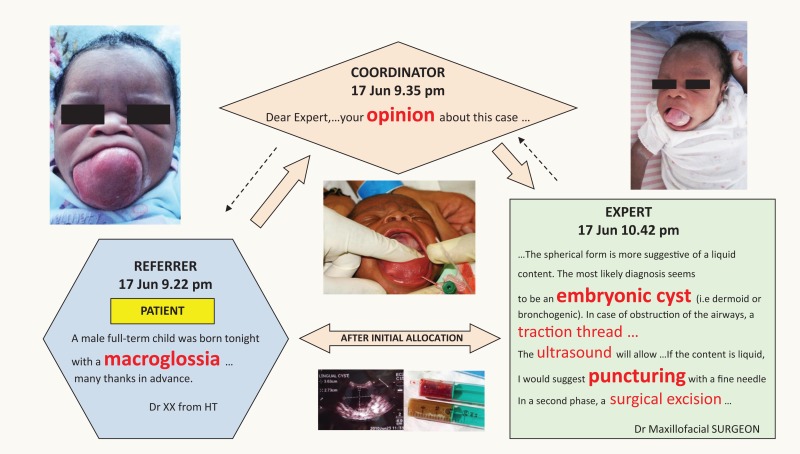

The aim of the present study was:

1. to review telemedicine activity in the first 4 years
2. to assess user satisfaction with the system.

## Materials and Methods

We conducted a retrospective analysis of all cases referred from April 2010 to March 2014. Information relating to the cases was extracted from the database of the tele-expertise system. Ethics permission was not required, because patient consent to access the data had been obtained and the work was a retrospective chart review conducted by the organization’s staff in accordance with its research policies.

We also carried out a survey of all users in December 2013. The survey contained 50 questions. These were closed-ended, multiple-choice, and scale type questions, and open-ended questions. The questions were established after literature research combined with qualitative data collection (in-depth interview and participating observation). The survey was tested on three referrers and three specialists, in English and in French. After the pilot testing, the survey was sent to all referrers and specialists registered in the database, regardless of whether or not they were active (i.e., had logged in and sent or answered cases). Versions of the survey were made available in French and English. Web-based software (https://www.surveymonkey.com/) was used for collecting the data.

Data were examined with the usual methods for quantitative analysis, while the results of the open-ended questions were processed in a qualitative way. The present paper reports a preliminary analysis of the survey results.

## Results

### Development of the network

**Figure 1:**
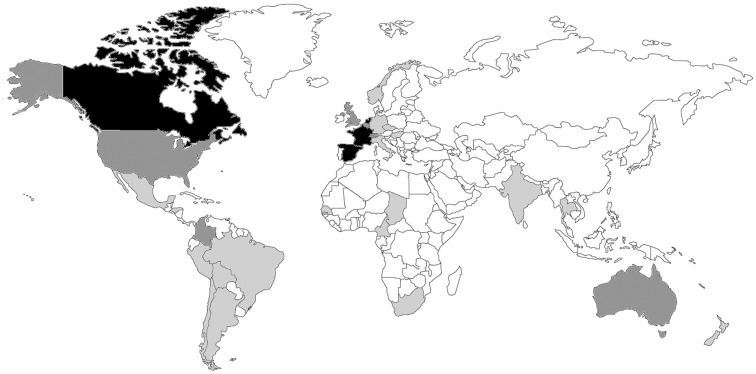
**Country of origin of the specialists (*n* = 269)**. The countries are shaded: light gray, <5 specialists; dark gray, 5–25 specialists; black, >25 specialists.

Over a 4-year period, the tele-expertise system evolved from separate, single-language telemedicine networks to an integrated, multilingual system. This encompassed:

300 field health workers from all MSF operational centers (French, Dutch, Belgium, Spanish, and Swiss)
250 volunteer specialists from all over the world (Figure 1). The specialists cover most of the medical and surgical specialties; 90% have direct MSF or field experience
9 case-coordinators, who are volunteers:
  1 in each language (English, French, Spanish);
  1 within each of the 5 MSF operational centers;
  1 for radiological cases
  2 software engineers (part-time).

### System performance

**Figure 2:**
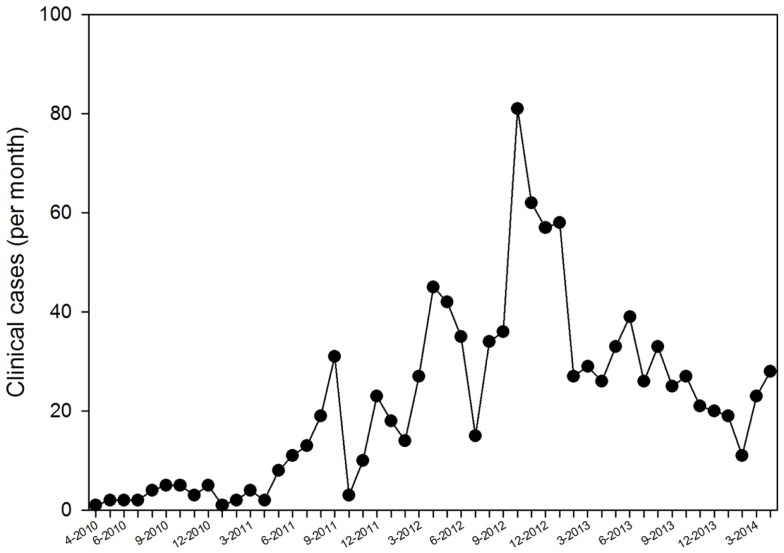
**Submission rate of clinical cases (*n* = 1067)**.

During the 4-year study period, the caseload rose in the first 2 years and subsequently stabilized at about 1–2 cases/day (Figure 2). The peak was mainly the result of radiology cases submitted from a single hospital in the Central African Republic, which had no radiological expertise available on-site. Fluctuations were mainly related to specific implementation episodes and to promotion in presentations to the organization’s staff.

**Figure 3:**
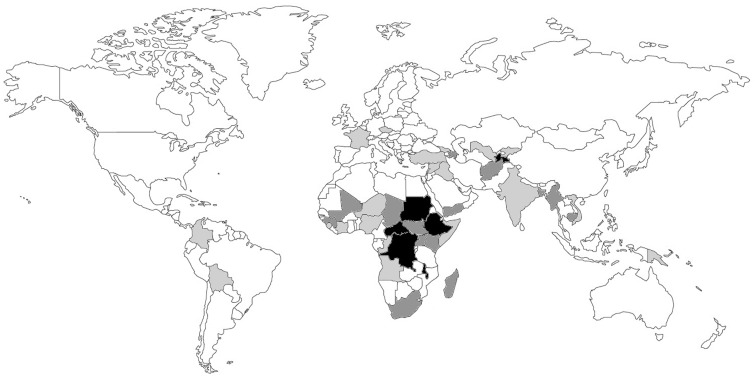
**Country of origin of the telemedicine cases (*n* = 1039)**. The countries are shaded: light gray, <5 cases; dark gray, 5–50 cases; black, >50 cases. The country of origin could not be determined in a small proportion of cases (1.2%).

A total of 1039 referrals were received from 41 countries (Figure 3), of which 89% were in English, 10% in French, and 1% in Spanish.

The majority of the case allocations were done by four case-coordinators (93%). The median delay in allocating a new case was 0.5 h (interquartile range, IQR 0.17, 1.9). The median delay in providing the first specialist response to the referrer was 5.3 h (IQR 1.8, 16.4).

**Figure 4:**
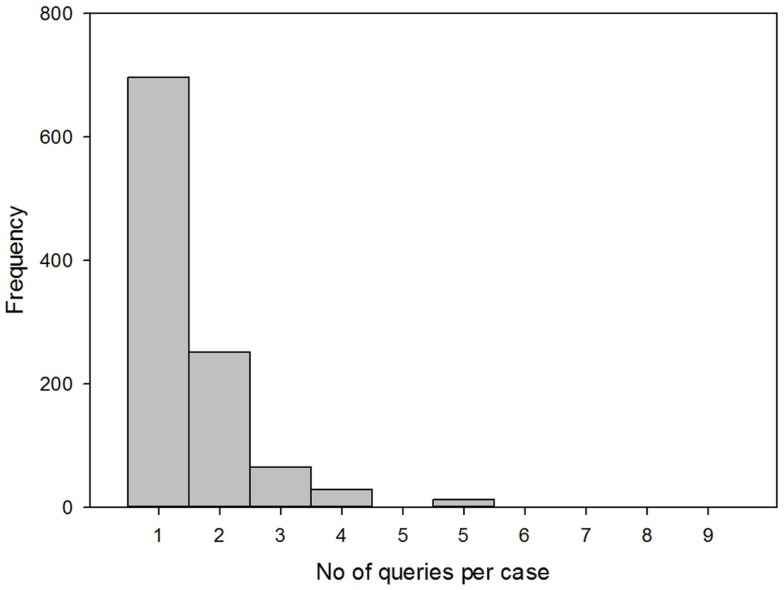
**Number of queries per case**.

Two-thirds of the cases (66%) required a single allocation (also known as a query) to produce a specialist response, while one-third of the cases required more than one allocation. The mean number of allocations per case was 1.51 (Figure 4). The median number of messages per case was 4 (IQR 3, 6).

### Case characteristics

The cases covered a very wide range of medical and surgical specialties (Table 1). The most common type of referral was for radiology (44%), which reflected the difficulties being experienced at a small number of hospitals in sub-Saharan Africa.

**Table 1:** **Types of queries**.

Main specialty	Sub-category	Queries
Allied health	Physiotherapy	1
Anesthesia	Anesthetics	1
Emergency medicine	Emergency medicine	1
General practice	General practice	1
Internal medicine	Internal medicine	2
Internal medicine	Cardiology	4
Internal medicine	Dermatology	72
Internal medicine	Endocrinology	6
Internal medicine	Gastroenterology	7
Internal medicine	Geriatrics	8
Internal medicine	Hematology	12
Internal medicine	Infectious diseases	139
Internal medicine	Intensive care	5
Internal medicine	Neurology	19
Internal medicine	Ophthalmology	6
Internal medicine	Renal	8
Internal medicine	Respiratory	14
Internal medicine	Sexual and reproductive health	1
Internal medicine	Tropical diseases	15
Internal medicine	Tropical medicine	4
Mental health	Psychiatry	7
Obstetrics and gynecology	O&G	33
Other	Other	2
Pediatrics	General	169
Pediatrics	Cardiology	3
Pediatrics	Infectious diseases	84
Pediatrics	Intensive care	26
Pediatrics	Neonatal	39
Pediatrics	Neurology	2
Pediatrics	Renal	1
Pathology	Microbiology	1
Radiology	Diagnostic	668
Surgery	General	24
Surgery	Abdominal	5
Surgery	ENT	24
Surgery	Max-Fac	10
Surgery	Neurosurgery	7
Surgery	Oncology	4
Surgery	Ophthalmology	49
Surgery	Orthopedics	32
Surgery	Plastic	8
Surgery	Thoracic	2
Total		1526

**Figure 5:**
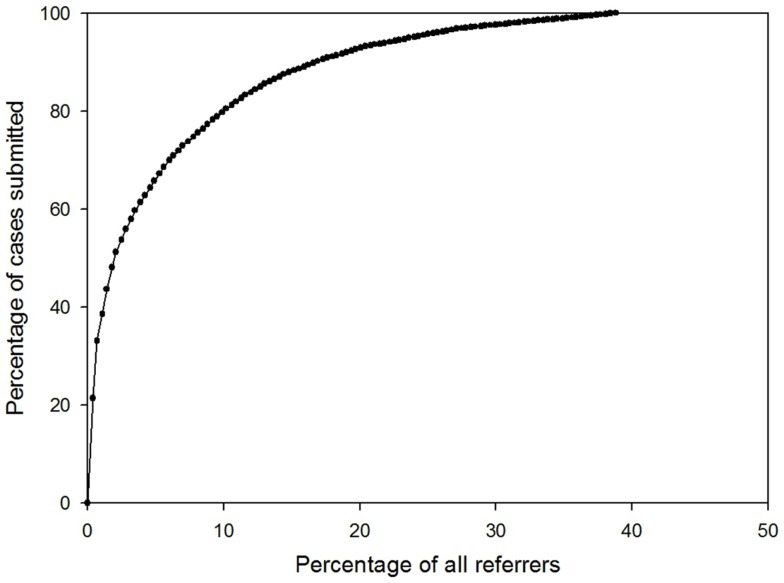
**Percentage of all referrers who had submitted a case**.

The majority of the cases were submitted by relatively few referrers (see Figure 5). For example, 80% of cases were submitted by only 10% of the referrers.

**Figure 6:**
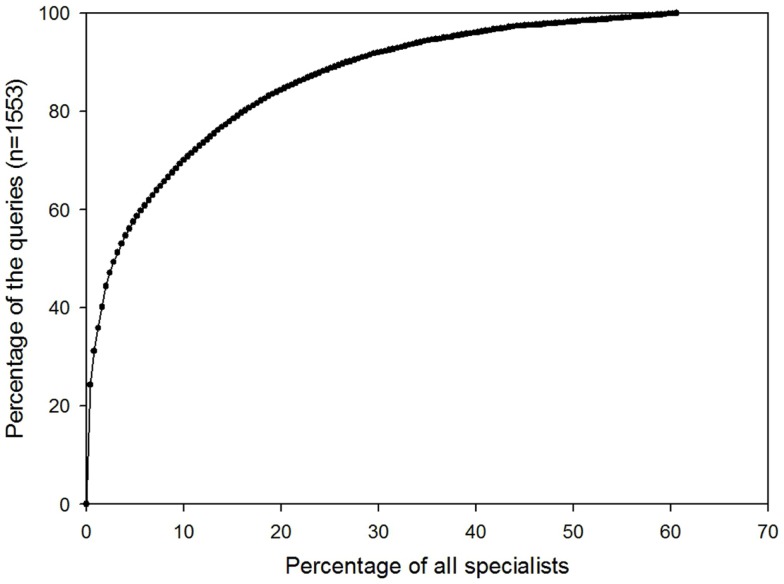
**Percentage of all specialists who had been sent a query**.

Similarly, the majority of queries were answered by relatively few specialists (see Figure 6). For example, 80% of queries were sent to only 16% of all specialists.

### Survey results

The survey was sent to 294 referrers and 254 specialists. Of these, 224 were considered as active users (41%). Out of the 548 users, 163 (30%) answered the survey. The survey was completed reasonably promptly by the majority of the respondents: 70% questionnaires were completed within 6 days. Responses from French and English users were analyzed together.

The survey results were generally positive (Tables 2A,B) demonstrating a high level of user participation, despite the questionnaire being rather long (50 questions).

**Table 2:** **(A) Specialist responses; (B) referrer responses**.

Question	Yes	No	Unknown	Total answered	Skipped	Percentage yes
**(A) SPECIALIST RESPONSES**						
Q28: Was the information supplied by the referrer adequate?	44	22	–	66	33	66.7
Q30: Was the referrer question clear?	59	5	–	64	35	92.2
Q34: Did the advice improve patient management?	30	2	35	67	32	44.8
Q35: Was there any educational benefit to the referrer?	75	1	–	76	23	98.7
	Average 3.20^a^					
Q36: Did the consultation have any value for you personally?	56	11	–	67	32	83.6
Q36: If yes, what kind of value was it?^b^
Mainly clinical	11	–	–	11	–	19.6^b^
Mainly educational	7	–	–	7	–	12.5
Both clinical and educational	30	–	–	30	–	53.6
Other	12	–	–	12	–	21.4
Q41: What is your satisfaction with the system?	Average 6.63^c^			73	26	–
Q43: Is there benefit in having access to specialized medicine in a low-resource setting?	Average 8.04^c^			82	17	–
Q44: Are referrers isolated in their practice?	Average 7.21^c^			81	18	–
Q45: Can the system help?
… Overall	Average 3.63^a^			71	28	–
… Feel less isolated	Average 3.65^a^			75	24	–
**(B) REFERRER RESPONSES**						
Q30: Did you find the advice helpful?	30	3	–	33	31	90.9
Q32: Was the answer appropriate/adapted to your field environment?	31	2	–	33	31	93.8
Q33: Did the advice improve patient management?	26	2	5	33	31	78.8
Q36: Was there any educational benefit to you (referrer)?	31^d^	2	–	33	31	93.9
	Average rating 2.94^a^					
Q43: Would you recommend the system to your colleagues?	33	1	–	34	30	97.1
Q44: What is your satisfaction with the system?	Average 7.61^c^			36	28	–
Q46: Is there benefit in having access to specialized medicine in a low-resource setting?	Average 8.27^c^			41	23	–
Q47: Are referrers isolated in their practice?	Average 6.76^c^			41	23	–
Q48: Can the system help?
… Overall	Average 3.65^a^			36	27	–
… Feel less isolated	Average 3.67^a^			37	29	–

*^a^Average rating: scale 1 = no, 2 = a little, 3 = moderately, 4 = a lot*.

*^b^Percentage of total answering yes (56)*.

*^c^Average rating: scale from 0 = not happy at all to 10 = extremely happy with it*.

*^d^Answer yes = categories 2–4 below*.

The main features of the users and their IT habits are shown in Table 3. Answers to questions related to satisfaction and the benefits of system use are shown in Tables 3 and 4. Although many users skipped this part of the survey, this was mainly because the questions could not be answered unless the respondent had actually used the tele-expertise system.

**Table 3:** **Main features of the user profile**.

	Referrer	Specialist
**USER PROFILE**		
Qualification	MD (74%) > nurse (19%)	MD (95%), an average of 17 years of expertise
Number of missions	More than 5	More than 5
Cumulative duration mission	1–5 years	<1 year
Job position/location	Medical team leader > medical coordinator	Teaching hospital > Public Health service – NGO > private sector
**INTERNET ISSUE**		
Internet access frequency	More than twice a day	Continuously
Quality of connection	Medium	High
Ability to send file attached	Easy if small	Easy whatever size
Type of connection mainly used	Wifi > Ethernet – Modem > Mobile	Wifi > Ethernet – Modem > Mobile
Device	Laptop > mobile > tablet	Laptop > mobile > tablet
Other networks used	Facebook > professional medical network > Twitter	Professional medical network > Facebook > Twitter
Involved in other telemedicine networks	No (80%)	No (77%)
Any concern in using telemedicine	No (76%)	No (78%)
**WEBSITE (telemed.msf.org)**		
Any briefing about the system?	No (60%)	Yes, sufficient (38%), Yes, not sufficient (12%)
User friendly	Yes (84%)	Yes (77%)
Self-explanatory	Yes (58%)	Yes (79%)
Problem with login or password	Never/sometimes > regularly (7%)	Never > sometimes > regularly (11%)
Problem with internet connection	Never/sometimes > regularly (12%)	Never (76%) > sometimes
Efficient assistance	Yes (80%)	Yes (75%)
**CASE ISSUE**		
How long did it take to write or answer a case?	10–20 min	More than 20 min
Did you deal with it offline or online?	Offline	Online
Was it difficult to find time to answer?	NA	No (66%)
Was information given to the patient about system	Yes (67%)	NA
Consent given	Orally (76%), but never written	NA
**DELAY TO SPECIALIST ANSWER**		
Desirable	<6 h	12–24 h
Acceptable	12–24 h	12–24 h
Was follow-up given by the referrer or received by the specialist	No (59%)	No (92%)
In your opinion, is follow-up?	Desirable > necessary > optional/mandatory	Desirable > necessary > mandatory (not optional)
In your opinion, when is the right time to give follow-up?	After 1 week	NA
**EXPERT ISSUE**		
Is volunteering the right status for experts?	NA	Yes (95%)
Should experts receive payment?	NA	No (95%)
How many cases could you reasonably answer?	NA	One per week > 3 per week
Would you answer cases for a non-MSF network?	NA	Yes (81%)

*For the yes/no questions, the value in brackets represents the majority response. For the multiple-choice questions, the value shown is the majority response*.

*NA, not applicable or not asked*.

**Table 4:** **Comparison of referrer and specialist opinions about the benefits and satisfaction with the system**.

Question	Referrer			Specialist		
Did the advice improve patient management?	Yes	No	Unknown	Yes	No	Unknown
	79%	6%	15%	45%	3%	52%
Was there any educational benefit to the referrer?	Average rating 2.94^a^			Average rating 3.20^a^		
What is your satisfaction with the system?	Average 7.61^b^			Average 6.63^b^		
Is there benefit in having access to specialized medicine in a low-resource setting?	Average 8.27^b^			Average 8.04^b^		
Are referrers isolated in their practice?	Average 6.76^b^			Average 7.21^b^		

*^a^Scale 1 = no, 2 = a little, 3 = moderately, 4 = a lot*.

*^b^Average rating: scale from 0 = not happy at all to 10 = extremely happy with it*.

A summary of the commonly-occurring referrer and specialist comments made in response to the open-ended questions are shown in Tables 5 and 6. The main concerns raised by referrers and specialists were the lack of support or promotion of system at headquarters’ level and the lack of feedback about patient follow-up.

**Table 5:** **Summary of referrer comments (open-ended questions)**.

	Number of comments
Lack of headquarters’ support in using the system	5
Satisfaction (e.g., “excellent,” “congratulations,” “thank you”)	4
Lack of promotion of the system	4
Reduced isolation of field doctors	2
Briefing should be improved	2
Proposal to use other technology (e.g., video, SMS)	2

**Table 6:** **Summary of specialist comments (open-ended questions)**.

	Number of comments
Lack of feedback about patient follow-up	9
No case received/frustration/disappointment	7
Satisfaction (e.g., “congratulations”)	2
Importance of field experience for giving a well-adapted answer	2

## Discussion

Médecins Sans Frontières has previously used both store-and-forward and real-time telemedicine (5, 6). Although the real-time telemedicine work was considered successful, the requirement for good quality Internet connections makes real-time telemedicine much more expensive than store-and-forward work. Cost is crucial in the humanitarian context or in places which have very few resources. Indeed, the consequences of wastage that would have little effect on health care in high income countries can have a profound impact in low-resource settings. Store-and-forward telemedicine certainly has disadvantages in comparison with real-time telemedicine – principally, the interaction between the parties is not as immediate – but it also has considerable advantages: it is cheaper and it is easier to organize. Thus in a low-resource setting, store-and-forward telemedicine is inherently more likely to be sustainable.

The present review shows that the experience of the MSF tele-expertise system is generally positive. At the time of writing, it is in its fifth year of operation, and as is well-known, many telemedicine projects fail to survive beyond their initial set-up phase (7). Another positive sign is that the referrers who sent cases continued to do so, an objective demonstration of their satisfaction with the system and its value to them.

Comments made by the volunteer specialists suggest that they were highly motivated and frequently expressed frustration about not getting enough cases. It is clear that the positive image of MSF worldwide has been a key factor in recruiting and keeping motivated our specialist volunteers.

The creation of the network, which was set up initially in a few months, is itself a kind of achievement. It reflects the global footprint of MSF, its considerable field expertise, and its multilingualism and multiculturalism: 550 users, 74 countries connected, and tele-expertise available in three languages from specialists with significant field experience.

Based on the user survey, it is clear that the tele-expertise system is easy to use and provides clinically useful diagnostic and management advice to clinicians in the field. The majority of referrers (79%) reported that the advice received via the system improved their management of the patient. In contrast, only about half of the specialists (45%) felt that the advice they had given would improve patient management while another half did not know/were not able to answer (unknown). The same phenomenon was reported in a recent survey of the users of the Swinfen telemedicine system (8). In the present study, the explanation may be that since many specialists had not answered any case, they were not in a position to comment on potential improvement.

Finally, if objective improvement in patent management remains to be demonstrated from the patient point of view, it is clear that there is a precious educational value for the referrers who take full advantage of expert advice and experience to assist them in overcoming their professional isolation.

### Limitations of the study

The main limitation of the present study is that it was retrospective, and there was no control system to compare it with. On the other hand, the survey questionnaires were completed by both users and non-users of the system, which reduces the bias inherent in surveys that are only completed by system users.

The response rate to the survey was not as high as would be expected in an online survey of doctors in industrialized countries, where response rates of 50–60% can be achieved. However, in the context of an online survey of telemedicine doctors in low-resource settings, the response rate was reasonable. For comparison, a previous survey of an HIV telemedicine network had a response rate of only 19% (9). The dangers of a low response rate are non-response bias (if the answers of respondents differ from the potential answers of those who did not respond) and response bias (if respondents tend to give answers that they believe that the questioner wants). Since we are not aware of the opinions of the non-responders, this may represent a potential source of bias in the present work.

### Lessons learned

The two main lessons learned concern the uneven pattern of system usage and the relative lack of referrer feedback:

#### Uneven pattern of usage

Although there are 550 registered users, only about half of them are active, i.e., have logged in and sent or answered cases. We believe that this is typical of large telemedicine systems of this type, but there appear to be few published reports for comparison. Furthermore, the distribution of activity among the active users was very uneven, e.g., 80% of cases were submitted by only 10% of the referrers, and 80% of queries were sent to only 16% of all specialists. This uneven pattern of usage may lead to the demotivation of specialists who agree to answer cases, but do not subsequently receive referrals. The uneven pattern of referrals may be a consequence of limited communication and promotion of the system by MSF, with little briefing of staff before their deployment to the field; both reflect a lack of political support to embrace telemedicine. In the future, positive attempts must be made to engage all users.

#### Lack of referrer feedback

Feedback from the referrer about patient follow-up is crucial for quality improvement and is necessary to keep the volunteer specialists informed about cases that they have advised on. The lack of feedback from referrers may also lead some specialists to lose interest in continued participation. Almost all specialists request follow-up after a teleconsultation (52% considered follow-up desirable and 47% considered it necessary or mandatory), and most referrers acknowledge a willingness to provide it. The reasons for the relative lack of follow-up data are probably not due to an unwillingness to provide it by the referrers. The stated reasons include a lack of time and a feeling that it was unnecessary. In addition, it is the nature of MSF operations in conflict zones and other resource-limited settings that patients are often seen in hospital, treated, and then disappear, not being available for subsequent follow-up to take place. Despite these practical difficulties, we have recently established a system by which follow-up requests are sent to the referrer automatically by email after a predetermined interval. This may improve the feedback in future.

## Conclusion

After 4 years of development, MSF has put into place a multilingual tele-expertise system to support workers in the field. User surveys confirm that the system provides helpful advice, which has a positive effect on patient outcomes. It is reliable and efficient. It improves patient management, has educational value for those involved, and reduces isolation for the referrers. Because of the size of the MSF organization, it is clear that there is potential for further organizational adoption. This will depend on political support from within the organization itself.

## Conflict of Interest Statement

The authors declare that the research was conducted in the absence of any commercial or financial relationships that could be construed as a potential conflict of interest.
